# Effective Perturbations of the Amplitude, Gating, and Hysteresis of *I*_K(DR)_ Caused by PT-2385, an HIF-2α Inhibitor

**DOI:** 10.3390/membranes11080636

**Published:** 2021-08-17

**Authors:** Hung-Tsung Hsiao, Guan-Ling Lu, Yen-Chin Liu, Sheng-Nan Wu

**Affiliations:** 1 Department of Anesthesiology, College of Medicine, National Cheng Kung University Hospital, National Cheng Kung University, Tainan City 70403, Taiwan; aneshsiao@yahoo.com.tw (H.-T.H.); r93443013@ntu.edu.tw (G.-L.L.); 2 Department of Anesthesiology, School of Post-Baccalaureate, College of Medicine, Kaohsiung Medical University, Kaohsiung City 80708, Taiwan; 3 Institute of Basic Medical Sciences, College of Medicine, National Cheng Kung University, Tainan City 70101, Taiwan; 4 Department of Physiology, College of Medicine, National Cheng Kung University, Tainan City 70101, Taiwan

**Keywords:** PT-2385, delayed-rectifier K^+^ current, current kinetics, voltage-dependent hysteresis, free energy, pituitary cell, glioma cell

## Abstract

PT-2385 is currently regarded as a potent and selective inhibitor of hypoxia-inducible factor-2α (HIF-2α), with potential antineoplastic activity. However, the membrane ion channels changed by this compound are obscure, although it is reasonable to assume that the compound might act on surface membrane before entering the cell´s interior. In this study, we intended to explore whether it and related compounds make any adjustments to the plasmalemmal ionic currents of pituitary tumor (GH_3_) cells and human 13-06-MG glioma cells. Cell exposure to PT-2385 suppressed the peak or late amplitude of delayed-rectifier K^+^ current (*I*_K(DR)_) in a time- and concentration-dependent manner, with *IC*_50_ values of 8.1 or 2.2 µM, respectively, while the *K*_D_ value in PT-2385-induced shortening in the slow component of *I*_K(DR)_ inactivation was estimated to be 2.9 µM. The PT-2385-mediated block of *I*_K(DR)_ in GH_3_ cells was little-affected by the further application of diazoxide, cilostazol, or sorafenib. Increasing PT-2385 concentrations shifted the steady-state inactivation curve of *I*_K(DR)_ towards a more hyperpolarized potential, with no change in the gating charge of the current, and also prolonged the time-dependent recovery of the *I*_K(DR)_ block. The hysteretic strength of *I*_K(DR)_ elicited by upright or inverted isosceles-triangular ramp voltage was decreased during exposure to PT-2385; meanwhile, the activation energy involved in the gating of *I*_K(DR)_ elicitation was noticeably raised in its presence. Alternatively, the presence of PT-2385 in human 13-06-MG glioma cells effectively decreased the amplitude of *I*_K(DR)_. Considering all of the experimental results together, the effects of PT-2385 on ionic currents demonstrated herein could be non-canonical and tend to be upstream of the inhibition of HIF-2α. This action therefore probably contributes to down-streaming mechanisms through the changes that it or other structurally resemblant compounds lead to in the perturbations of the functional activities of pituitary cells or neoplastic astrocytes, in the case that in vivo observations occur.

## 1. Introduction

Hypoxia-inducible factor-1α (HIF-1α) has been reported to be expressed in pituitary tumors [1,2,3,4,5,6,7,8,9,10]. Alternatively, HIF-2α has also been reported to be expressed in pituitary tumors [11]. HIF-2α antagonists such as PT-2385 ((S)-3-((2,2-difluoro-1-hydroxy-7-(methylsulfonyl)-2,3-dihydro-1*H*-inden-4-yl)oxy)-5-fluorobenzoniltrile) are increasingly being proposed as a potential effective treatment of choice for HIF-2α-related tumors and metabolic disorders [10,11,12,13,14,15,16,17,18,19,20,21,22,23,24,25,26]. As a potent inhibitor of HIF-2α, PT-2385 was thought to selectively disrupt the heterodimerization of HIF-2α with HIF-1β [12,14,21]. It is very potent in impeding cancer cell growth, proliferation, and tumor angiogenesis, particularly in clear-cell renal cell carcinoma [12,13,14,20,21,22]. A report has recently revealed that roxadustat (FG-4592), a small molecule recognized to be an inhibitor of HIF prolyl hydroxylase, was effective in perturbing the amplitude and gating of voltage-gated K^+^ currents [27]. However, whether or how PT-2385 is capable of modulating the strength of plasmalemmal K^+^ currents is uncertain.

Voltage-gated K^+^ (K_V_) channels are well-recognized as having essential roles in determining the membrane excitability associated with delayed-rectifier K_V_ channels, for example K_V_3 (KCNC) or K_V_2 (KCNB) channels, and they are expressed ubiquitously in neuroendocrine or endocrine cells [28,29,30,31,32,33,34]. A cause-and-effect relationship regarding the activity of K_V_3 or K_V_2 channels and the magnitude of delayed-rectifier K^+^ currents (*I*_K(DR)_) is correlated with action potential firing in many cell types and has increasingly been demonstrated [29,30,31,32,35,36,37,38,39,40,41,42,43,44]. K_V_ channels of the K_V_3.1–K_V_3.2 types have also been regarded to be the main determinants of *I*_K(DR)_ identified in pituitary cells, including pituitary tumor (GH_3_) cells [28,30,31]. The biophysical properties of *I*_K(DR)_ in GH_3_ cells are unique in being manifested by a positively shifted voltage dependency as well as by a rapid deactivation rate [27,30,31,35,41,45]. The activity of HIF-1α has recently been reported to regulate the proliferation and invasiveness of oral cancer cells through the Kv3.4 channel [23]. Alternatively, PT-2385 was reported to have rapid effects on the impairment of hypoxic ventilator control [46]. Our understanding of whether PT-2385 or other related compounds cause any adjustments with regard to the amplitude, gating, and hysteresis of these types of K^+^ currents (e.g., *I*_K(DR)_) remains largely incomplete, although recent studies have demonstrated a possible link between the expression of HIF and the regulation of K_V_-channel activity [23,47,48,49,50].

In view of the foregoing considerations, the following attempts were undertaken to determine how PT-2385 and other relevant compounds could result in adjustments to the ionic currents in pituitary tumor (GH_3_) cells. Moreover, some researchers have performed studies to demonstrate the effectiveness of this compound in suppressing the growth and invasiveness of gliomas [18,19]. Therefore, we decided to investigate the possible actions of PT-2385 on *I*_K(DR)_ in the GH_3_ cells of pituitary and human 13-06-MG glioma cells. Findings from the present observations tempt us to reflect that the *I*_K(DR)_ inherent in different cell types could be an additional non-canonical target through which PT-2385 can act to influence the functional activities of the cells involved.

## 2. Materials and Methods

### 2.1. Chemicals and Solutions

PT-2385 (PT, PT2385, (S)-3-((2,2-difluoro-1-hydroxy-7-(methylsulfonyl)-2,3-dihydro-1*H*-inden-4-yl)oxy)-5-fluorobenzoniltrile, C_17_H_12_F_3_NO_4_S) was acquired from MedChemExpress (Asia Bioscience, Taipei, Taiwan). Diazoxide (Diaz), tetrodotoxin (TTX), and tetraethylammonium chloride (TEA) were from Sigma-Aldrich (Merck, Taipei, Taiwan). Sorafenib (SOR) was purchased from Selleck (Asia Bioscience, Taipei, Taiwan), and cilostazol (Cil) from Tocris (Union Biomed, Taipei, Taiwan). PT-2385 was dissolved as 20 mM stock solution in dimethyl sulfoxide and was diluted by extracellular solution to achieve the final concentrations. Unless stated otherwise, we obtained fetal bovine and calf serum, L-glutamine, trypsin/EDTA, and the culture media (e.g., Ham’s F12-12 medium) from HyClone^TM^ (Thermo Fisher; Level Biotech, Tainan, Taiwan). Additionally, other chemicals were acquired at the best available quality, of analytical grade.

The normal Tyrode’s solution contained (in mM): NaCl 136.5, KCl 5.4, CaCl_2_ 1.8, MgCl_2_ 0.53, glucose 5.5, and 4-(2-hydroxyethyl)piperazine-1-ethanesulfonic acid (HEPES) 5.5, and the pH was adjusted to 7.4 with NaOH. For measurements of *I*_K(DR)_ across the cell membrane, the cells were placed in Ca^2+^-free Tyrode’s solution in order to preclude the contamination of voltage-gated Ca^2+^ currents and Ca^2+^-activated K^+^ currents. In whole-cell recordings, we filled the pipet with internal solution, which contained: KH_2_PO_4_ 1, K-aspartate 140, MgCl_2_ 1, Na_2_ATP 3, EGTA 0.1, Na_2_GTP 0.1, and HEPES 5 (in mM), and the pH was adjusted to 7.4 with KOH. To measure *I*_K(DR)_ in 13-06-MG glioma cells, the high K^+^-bathing solution contained: KCl 145, MgCl_2_ 0.53, and HEPES-KOH 5 (in mM and pH 7.4). All solutions were prepared by de-mineralized water with the water purification system Milli-Q^®^ (Merck, Tainan, Taiwan). During experimental days, the bathing or backfilling solution and culture medium were filtered by using an Acrodisc^®^ syringe filter with 0.2-µm Supor^®^ nylon membrane (Pall; Bio-Check, Tainan, Taiwan).

### 2.2. Cell Preparations

The preparation of GH_3_ or 13-06-MG is reported in the Supplementary Materials. References [51,52] are cited in the Supplementary Materials.

### 2.3. Electrophysiological Measurements

GH_3_ or 13-06-MG cells were harvested and rapidly transferred to a customized chamber shortly before the electrical recordings. The chamber was positioned on the stage of an inverted microscope. Cells were kept for immersion in normal Tyrode’s solution at 20–25 °C; the composition of this solution is described above. Patch-clamp recordings were undertaken under whole-cell mode with either an RK-400 (Biologic, Echirolles, France) or an AxoClamp 2B amplifier (Molecular Devices; Kim Forest, Tainan, Taiwan) [52,53]. Patch electrodes with tip resistance of 3–5 MΩ were made from Kimax-51 capillaries (#34500 (1.5–1.8 mm in outer diameter); Dogger, Tainan, Taiwan), using either a PP-830 vertical puller (Narishige, Tokyo, Japan) or a P-97 horizontal puller (Sutter, Novato, CA), and their tips were then fire-polished with MF-83 microforge (Narishige). The signals, which comprised voltages and current tracings, were stored online at 10 kHz in a touchscreen computer (ASUSPRO-BU401LG, ASUS, Tainan, Taiwan) equipped with Digidata 1440A interface (Molecular Devices), controlled by pCLAMP 10.7 software (Molecular Devices). The potentials were revised for the liquid–liquid junction potential that appeared when the composition of the pipette solution was different from the solution of the bath.

### 2.4. Data Analyses

To determine the concentration-dependent inhibitory effect of PT-2385 on *I*_K(DR)_, ionic current (i.e., *I*_K(DR)_) was evoked by a depolarizing pulse (1-s duration) from −50 to +50 mV, and current amplitudes (i.e., peak or late *I*_K(DR)_) taken with or without the addition of varying PT-2385 concentrations were measured at the beginning or end of test potential. The *I*_K(DR)_ in the control period (i.e., PT-2385 was not present) was considered to be 1.0, and the values obtained with different concentrations of PT-2385 were then compared and analyzed. The PT-2385 concentration yielding a 50% reduction (i.e., *IC*_50_) of peak or late *I*_K(DR)_ was evaluated with goodness-of-fit assessments by using the 3-parameter logistic model (i.e., a modified form of sigmoidal Hill equation). That is,

(1) $$\mathrm{Relative}\;\mathrm{amplitude}=\frac{{\left[PT\right]}^{-n_{H}}\times \left(1-a\right)}{{\left[PT\right]}^{-n_{H}}+IC_{50}^{-n_{H}}}+a$$

where [*PT*] is the PT-2385 concentration applied, *IC*_50_ is the PT-2385 concentration required for 50% inhibition of peak or late *I*_K(DR)_, and *n_H_* is the Hill coefficient; moreover, maximal inhibition (i.e., 1 − *a*) was approximated from the equation. This equation converged reliably to produce the best-fit line and parameter estimates.

The relationships either between the normalized amplitude and the conditioning potential (i.e., the quasi-steady-state inactivation curve of *I*_K(DR)_) or between the instantaneous current and the upsloping end of ITRV (i.e., instantaneous current-voltage [*I-V*] relationship of the current) were constructed and then fitted with a Boltzmann function in the following form:

(2) $$I=\frac{I_{max}}{1+exp\left[\frac{\pm \left(V-V_{1/2}\right)qF}{RT}\right]}$$

In this equation, *I_max_* is the maximal amplitude of *I*_K(DR)_ activated in response to step or ramp depolarization, *V*_1/2_ is the voltage at which there is half-maximal inactivation or activation of the current, *q* is the apparent gating charge, *F* is the Faraday constant, *R* is the ideal gas constant, and *T* is the absolute temperature.

The free energy involved in the gating of *I*_K(DR)_ (∆G_0_) was evaluated on the assumption that there is a 2-state (i.e., in an equilibrium between closed (resting) and open states) gating model existing in the K_V_ channel. The ∆*G*_0_ for *I*_K(DR)_ activation at 0 mV would be equal to *q* × *F* × *V*_1/2_ [54,55,56,57]. The standard errors of ∆*G*_0_ (i.e., $\sigma_{qFV_{1/2}}$) were calculated according to:

(3) $$\sigma_{qFV_{1/2}}=F\times \sqrt{V_{1/2}^{2}\sigma_{q}^{2}+q^{2}\sigma_{V_{1/2}}^{2}}$$

where *σ_q_* or $\sigma_{V_{1/2}}$ represents the standard error in *q* or *V*_1/2_, respectively.

### 2.5. Statistical Analyses

Linear or nonlinear curve fitting to experimental data sets presented herein was performed using either the Microsoft Solver function embedded in Excel^®^ 2016 (Microsoft) or the OriginPro 2021 program (OriginLab^®^; Scientific Formosa, Kaohsiung, Taiwan) [58]. Values of experimental measurements were presented as the mean ± standard error of mean (SEM) and sample sizes (n) indicative of the cell number which the experimental results were collected from, and the error bars were hence plotted as the SEM. The Student’s *t* test (paired or unpaired) was initially employed; however, as the statistical difference among different groups was necessarily evaluated, we performed either analysis of variance (ANOVA)-1 or ANOVA-2 (with or without repeated measure) followed by Duncan’s post hoc test. Differences of mean values between groups were considered significant at *p* < 0.05.

## 3. Results

### 3.1. Effect of PT-2385 on Delayed-Rectifier K^+^ Current (I_K(DR)_) Measured from Pituitary Tumor (GH_3_) Cells

**Figure 1:**
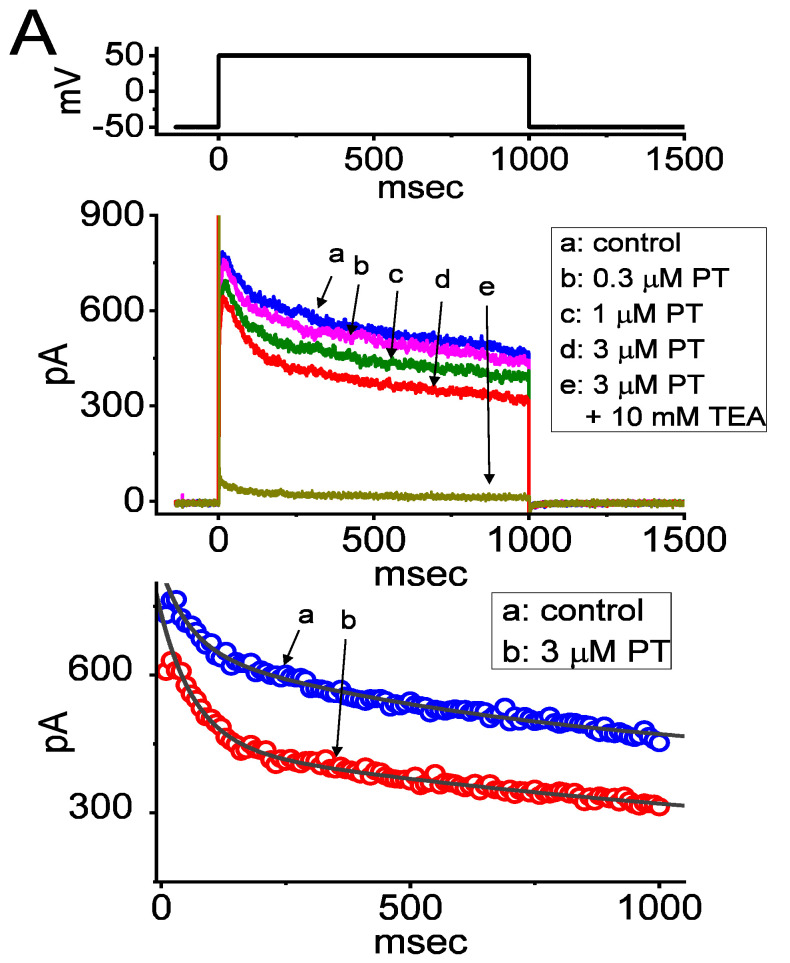

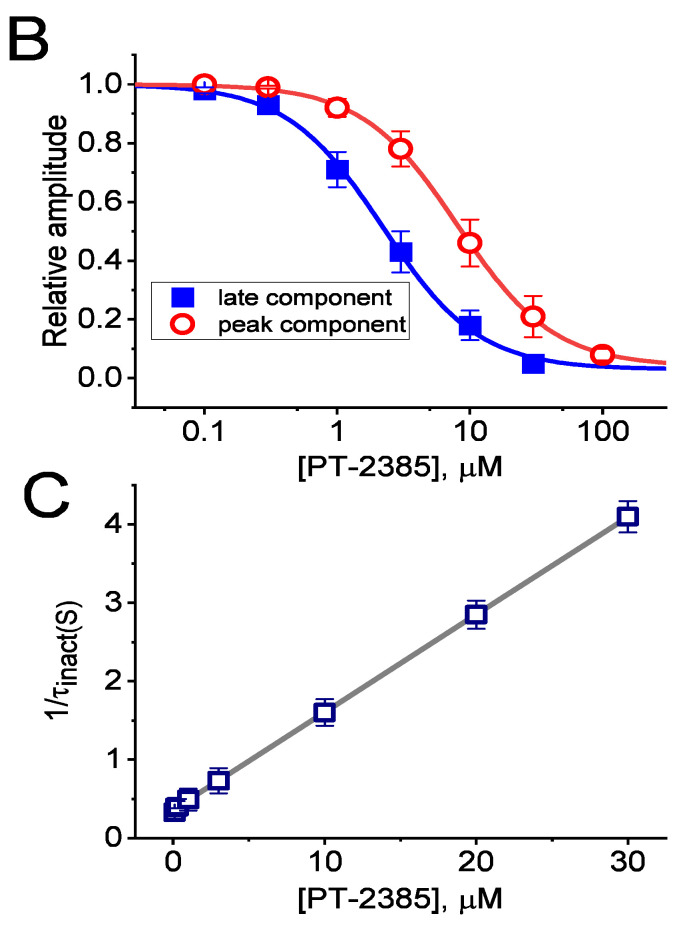
Inhibitory effect of PT-2385 (PT) on delayed-rectifier K^+^ current (*I*_K(DR)_) measured from pituitary tumor (GH_3_) cells. Whole-cell patch-clamp experiments were undertaken via experimented cells in Ca^2+^-free Tyrode’s solution containing 1 µM tetrodotoxin (TTX) and 0.5 mM CdCl_2_, while the recording pipet was filled with K^+^-containing solution. In the upper part of (**A**), representative *I*_K(DR)_ tracings were obtained for the control (i.e., PT-2385 was not present, (a)), and during cell exposure to 0.3 µM PT-2385 (b), 1 µM PT-2385 (c), 3 µM PT-2385 (d), or 3 µM PT-2385 plus 10 mM tetraethylammonium chloride (TEA). The voltage-clamp profile is illustrated in the uppermost part. In the lower part of (**A**), the time courses of current inactivation in the absence (a) and presence (b) of 3 µM PT-2385 were well approximated by a 2-exponential decay (indicated in gray smooth line). The values of fast or slow components (i.e., τ_inact(S)_) in the inactivation time constants of *I*_K(DR)_ obtained in the absence and presence of 3 µM PT-2385 were 63 or 1514 msec, and 61 or 786 msec, respectively. (**B**) The concentration–response curve of PT-2385-induced block of peak or late *I*_K(DR)_ occurring in GH_3_ cells. The continuous line represents the best fit to the modified Hill equation, as elaborated in the Materials and Methods. The *IC*_50_ values for PT-2385-induced inhibition of peak or late *I*_K(DR)_ were reliably estimated to be 8.1 or 2.2 µM, respectively. Each point represents the mean ± SEM (*n* = 9–12). (**C**) Relationship of the PT-2385 concentration as a function of the slow component in the inactivation rate constant (1/τ_inact(S)_) (mean ± SEM; *n* = 8 for each point). Of note, the value of 1/τ_inact(S)_ is directly proportional to the PT-2385 concentration. Based on a minimal reaction scheme elaborated in the text, the value of *k*_+1_^*^ or *k*_−1_ was estimated to be 0.124 s^−1^µM^−1^ or 0.362 s^−1^, respectively; hence, the *K*_D_ value (*k*_−1_/*k*_+1_^*^, i.e., dissociation constant) turned out to be 0.036 µM (or 36 nM), a value which is closely similar to the *IC*_50_ value required for its inhibitory effect on late *I*_K(DR)_.

In this study, we initially exploited the whole-cell configuration of standard patch-clamp technique to assess the perturbations of PT-2385 in the ionic currents of GH_3_ cells. In order to record the currents of *I*_K(DR)_, cells were bathed in Ca^2+^-free Tyrode’s solution containing 0.5 mM CdCl_2_ and 1 µM tetrodotoxin (TTX) while the recording pipet was backfilled with K^+^-containing internal solution. TTX or CdCl_2_ was used to block voltage-gated Na^+^ or Ca^2+^ currents, respectively. As the whole-cell mode was established, the examined cell was maintained at the potential of −50 mV in voltage clamp mode and the depolarizing step was subsequently applied (1-s duration) to +50 mV for the elicitation of *I*_K(DR)_ [27,31,32,59]. Of interest, 1 min after cell exposure to PT-2385, the amplitude of *I*_K(DR)_ evoked by the 1-s-long step depolarization from –50 to +50 mV became progressively decreased (Figure 1A). For example, at the level of +50 mV, the addition of 3 µM PT-2385 was found to diminish the peak or late amplitude of *I*_K(DR)_ to 609 ± 63 or 318 ± 41 pA (*n* = 8, *p* < 0.05), respectively, from control values of 818 ± 72 or 529 ± 51 pA (*n* = 8). After washout of the agent, the peak or late amplitude of the current returned to 807 ± 69 or 517 ± 48 pA (*n* = 8, *p* < 0.05). In the continued presence of 3 µM PT-2385, the further addition of 10 mM tetraethylammonium chloride (TEA) almost fully abolished current amplitude. Additionally, in the presence of 3 µM PT-2385, the slow-component value in the inactivation time constant (τ_inact(S)_) of *I*_K(DR)_ activated by a 1-s depolarizing pulse to +50 mV was rapidly decreased to 786 ± 119 msec (*n* = 8, *p* < 0.05) from a control value of 1514 ± 217 msec (*n* = 8); however, a minimal change in fast component of current inactivation was seen in its presence. The results strongly suggest that PT-2385-mediated inhibition of *I*_K(DR)_ in GH_3_ cells is accompanied by an enhancement of the current inactivation rate (Figure 1A).

The correlation between the concentration of PT-2385 and the relative amplitude of peak or late *I*_K(DR)_ was further determined and established. In these experiments, we held the examined cell in a voltage clamp at −50 mV, and the depolarizing pulse (1-s duration) to +50 mV was applied, while current amplitudes in the presence of varying PT-2385 concentrations were taken at the start or end-pulse of step depolarization. As illustrated in Figure 1B, the presence of various concentrations of PT-2385 differentially suppressed the peak and late amplitudes of *I*_K(DR)_ in a concentration-dependent manner. By virtue of a non-linear least-squares fit to the experimental data, the value of the *IC*_50_ (i.e., concentration required from a half-maximal inhibition) for the inhibitory effect of PT-2385 on peak or late *I*_K(DR)_ was yielded as 8.1 or 2.2 µM, respectively. These results reflect that PT-2385 can exercise a depressant action on *I*_K(DR)_ in these cells, and that the late component of *I*_K(DR)_ activated by step depolarization was subject to being inhibited to a higher extent than the peak (or transient) component of the current.

### 3.2. Kinetic Estimate of I_K(DR)_ Block by PT-2385

During cell exposure to PT-2385, apart from the decreased *I*_K(DR)_ amplitude, the inactivation rate of the current responding to step depolarization tended to become raised. In this regard, we extended the study to estimate the inactivation kinetics of PT-2385-mediated inhibition of *I*_K(DR)_ activated by a long depolarizing pulse in situations where cells were exposed to varying PT-2385 concentrations. The concentration dependence of the *I*_K(DR)_-inactivation rate caused by adding this compound was constructed and is hence illustrated in Figure 1C. The experimental observations demonstrated that its effects on *I*_K(DR)_ resulted in a concentration-dependent increase in the rate of current inactivation. Consequently, the effect on *I*_K(DR)_ identified in GH_3_ cells can be reasonably explained by a state-dependent blocking mechanism through which this compound is allowed to preferentially bind to the open or open-inactivated state of the K_V_ channels. The reaction scheme for this viewpoint is thus simply

$$C\begin{array}{c} \overset{\alpha }{\to }\\ \underset{\beta }{\leftarrow } \end{array}O\begin{array}{c} \overset{k_{+1}^{*}\cdot \left[B\right]}{\to }\\ \underset{\;k_{-1\;}}{\leftarrow } \end{array}\;O\cdot \left[B\right]$$

Alternatively, the dynamical system yielding three equations becomes

(4) $$\frac{dC}{dt}=-\alpha \times C+\beta \times O$$

(5) $$\frac{dO}{dt}=\alpha \times C+k_{-1}\times O\cdot \left[B\right]-O\times \left(\beta +k_{+1}^{*}\cdot \left[B\right]\right)$$

(6) $$\frac{dO\cdot \left[B\right]}{dt}=-k_{-1}\times O\cdot \left[B\right]+k_{+1}^{*}\cdot \left[B\right]\times O$$

where *α* or *β* is called the kinetic constant for the opening or closing of the K_V_ channel, respectively; *k*_+1_^*^ or *k*_−1_ is that for blocking (i.e., depending on the PT-2385 concentration) or unblocking by adding PT-2385; [B] is the PT-2385 concentration used; and, C, O, or O·[B] indicates the closed (or resting), open, or open-blocked state of the K_V_ channel, respectively.

The blocking (forward, *k*_+1_^*^) or unblocking (backward, *k*_−1_) rate constant was estimated based on the slow component in the inactivation time constants (τ_inact(S)_) of *I*_K(DR)_ during cell exposure to varying PT-2385 concentrations (Figure 1C). The relationship can be satisfied by

(7) $$\frac{1}{\tau_{inact\left(S\right)}}=k_{+1}^{*}\times \left[B\right]+k_{-1}$$

In this equation, *k*_+1_^*^ or *k*_−1_ was respectively derived from the slope or from the *y*-axis intercept at *[B]* = 0 (i.e., the point at which the line crosses the y-axis) of the linear regression at which the reciprocal time constants of the current (1/*τ_inact(S)_*) versus the PT-2385 concentration was interpolated. The resultant relationship between 1/*τ_inact(S)_* and [B] was found in the straight line through experimental data points with a correlation coefficient of 0.95 (Figure 1C). The blocking or unblocking rate constant was thereafter yielded to be 0.124 s^−1^µM^−1^ or 0.362 s^−1^, respectively. According to the evolving rate constants, dividing *k*_−1_ by *k*_+1_^*^ gave a dissociation constant (*K*_D_) of 2.9 µM during cell exposure to PT-2385, a value that was noted to be close to the effective *IC*_50_ (2.2. µM) of this compound needed for inhibition of late *I*_K(DR)_.

### 3.3. Comparison among the Effects of PT-2385 (PT), PT-2385 Plus Diazoxide (Diaz), and PT-2385 Plus Cilostazol (Cil), or PT-2385 Plus Sorafenib (SOR) on the Amplitude of I_K(DR)_

**Figure 2:**
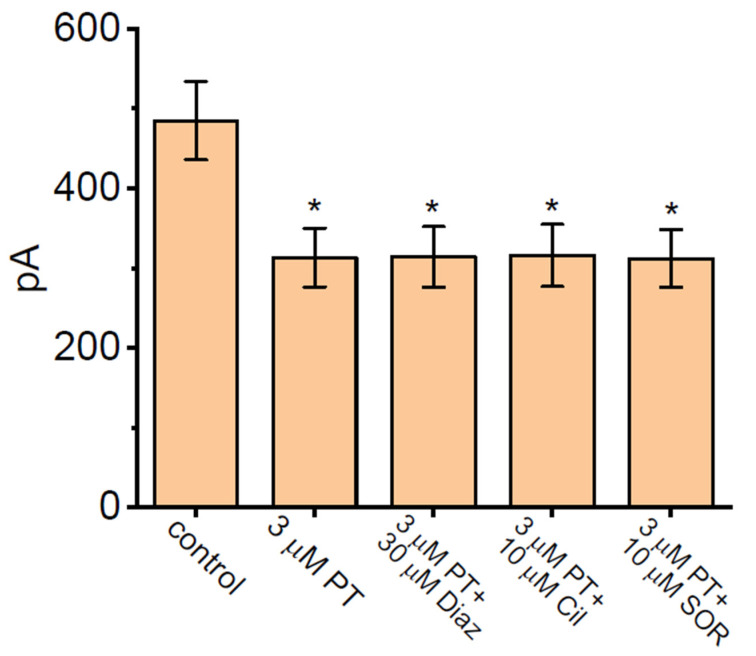
Comparisons among the effects of PT-2385 (PT), PT-2385 plus diazoxide (Diaz), PT-2385 plus cilostazol (Cil), and PT-2385 plus sorafenib (SOR) on *I*_K(DR)_ amplitude in GH_3_ cells (mean ± SEM; *n* = 7 for each bar). The whole experiments were performed in cells bathed in Ca^2+^-free Tyrode’s solution and the recording pipet was filled with K^+^-enriched internal solution. The *I*_K(DR)_ amplitude activated by a 1-s depolarizing step from −50 to +50 mV was taken at the end-pulse of command potential. In this set of experiments on PT-2385 plus tested compounds (e.g., Diaz, Cil, and SOR), each tested compound was subsequently added to the bath in the continued presence of PT-2385 (3 µM). * Significantly different from control (*p* < 0.05).

We continued to evaluate and then compare the effect of PT-2385 and PT-2385 plus different related compounds (e.g., Diaz, Cil, SOR) on *I*_K(DR)_ magnitude. As summarized in Figure 2, the amplitude of *I*_K(DR)_ was effectively inhibited by the addition of 3 µM PT-2385. However, as cells were continually exposed to PT-2385 (3 µM), the subsequent addition of either diazoxide (Diaz, 30 µM), cilostazol (Cil, 10 µM), or sorafenib (SOR, 10 µM) did not result in any further modifications to the PT-2385-mediated block of peak or late *I*_K(DR)_. Diaz or Cil has been reported to activate ATP-sensitive K^+^ (K_ATP_) channels or large-conductance Ca^2+^-activated K^+^ (BK_Ca_) channels, respectively [34,60,61], while SOR was previously recognized to suppress the activity of tyrosine kinase [62]. Additionally, the presence of Diaz (30 µM), Cil (10 µM), or SOR (10 µM) alone was not noted to have significant effects on the *I*_K(DR)_ amplitude observed in GH_3_ cells. Under this circumstance, it is reasonable to assume that the PT-2835-mediated block of *I*_K(DR)_ in GH_3_ cells tends to be independent of either the inhibition of K_ATP_ or BK_Ca_ channels, or the stimulation of tyrosine-kinase activity.

### 3.4. Inhibitory Effect of PT-2385 on Mean Current versus Voltage (I-V) Relationship of I_K(DR)_

**Figure 3:**
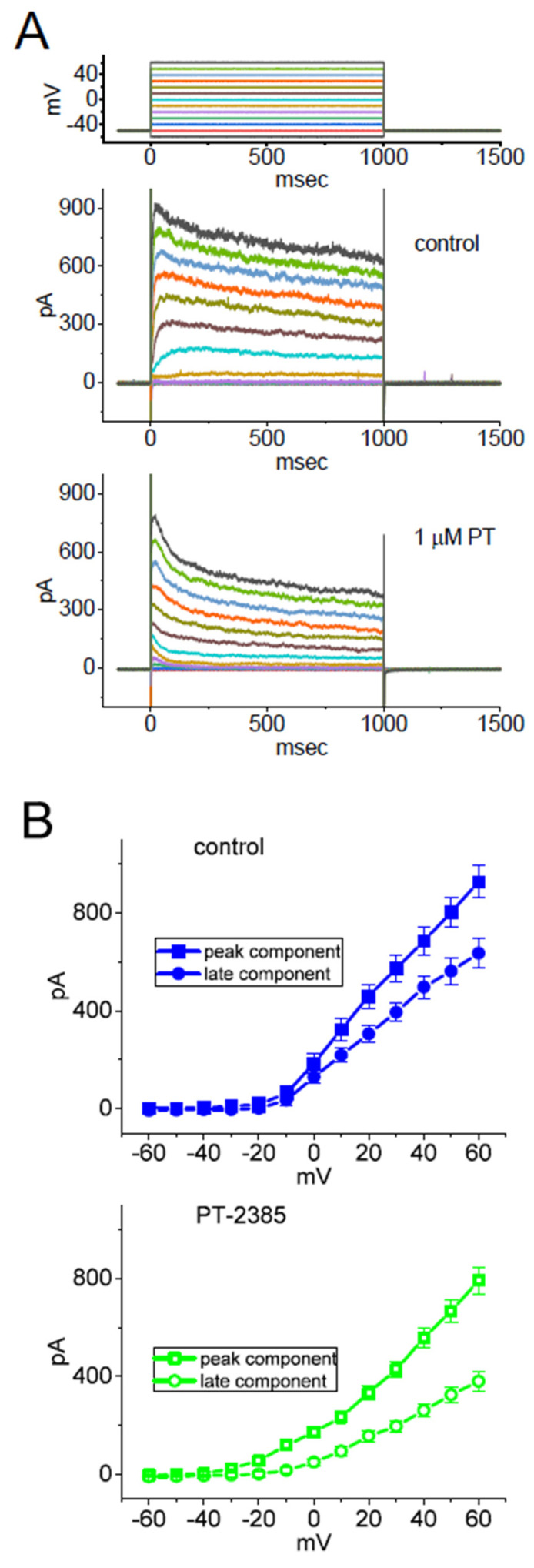
Effect of PT-2385 on mean the current versus voltage (*I-V*) relationship of *I*_K(DR)_ in GH_3_ cells. In these experiments, cell membrane potentials were held at −50 mV, and the depolarizing command pulse to a series of voltage steps (1-s duration) ranging between −60 and +50 mV in 10-mV increments (as indicated in the uppermost of (**A**)) was thereafter applied to evoke the currents. (**A**) Representative current traces in the control period (upper image) and during exposure to 1 µM PT-2385 (lower image). The current traces indicated in different colors with or without the presence of PT-2385 correspond to the potential ones in the uppermost part of (**A**). (**B**) Mean *I-V* relationships of *I*_K(DR)_ taken before (upper image, blue-filled symbols) or after (lower image, green open symbols) 1 µM PT-2385. The experimental data points in (**B**) were collected at the start (square symbols) or end-pulse (circle symbols) of the 1-s depolarizing voltage command. Each point represents the mean ± SEM (*n* = 8).

In another separate set of experiments, we voltage-clamped at –50 mV and then applied a series of command voltage pulses (1-s duration) from –60 to +50 mV in 10 mV increments to the examined GH_3_ cells. Under this voltage-clamp protocol, a family of *I*_K(DR)_ was robustly elicited, and the currents were manifested by an outwardly directed rectifying property with a small relaxation in the time course of current inactivation [31,41,45,59,62]. Of note, after 1 min of cell exposure to 1 µM PT-2385, the *I*_K(DR)_ magnitude became reduced, particularly at the potentials ranging between 0 and +50 mV (Figure 3A). Figure 3B respectively illustrates the mean *I-V* relationships of *I*_K(DR)_ measured at the start (initial peak) and end-pulse (late or sustained) of each potential obtained in the control period (upper panel) or during exposure to 1 µM PT-2385 (lower panel). For example, at the level of +50 mV, PT-2385 at a concentration of 1 µM led to decreased peak amplitude from 803 ± 62 to 667 ± 46 pA (*n* = 8, *p* < 0.05), while, at the same level of voltage pulse, this compound at the same concentration reduced the late *I*_K(DR)_ from 635 ± 61 to 381 ± 41 pA (*n* = 8, *p* < 0.05). After the agent was removed, the peak or late amplitude of *I*_K(DR)_ was back to 786 ± 58 or 611 ± 57 pA (*n* = 7, *p* < 0.05). It is conceivable, therefore, that the potency of the PT-2385-mediated block of late or sustained *I*_K(DR)_ activated by different step depolarizations (i.e., the potentials ranging between 0 and −50 mV) was higher than that for its blocking on the instantaneous peak component of the current.

### 3.5. The Steady-State Inactivation Curve of I_K(DR)_ during Exposure to PT-2385

**Figure 4:**
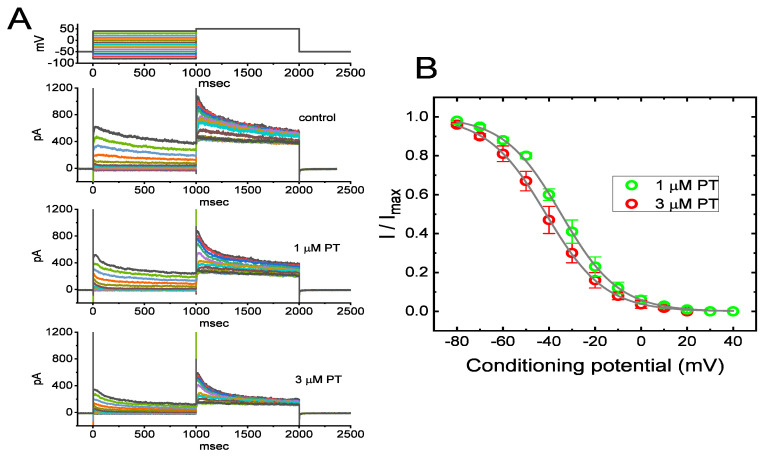
Effect of PT-2385 on the quasi-steady-state inactivation curve of *I*_K(DR)_ in GH_3_ cells. These experiments were undertaken using a 2-step voltage-clamp protocol (as indicated in the inset). (**A**) Representative *I*_K(DR)_ traces obtained in the control (i.e., PT-2385 was not present, upper image) and in the presence of 1 µM PT-2385 (middle image) or 3 µM PT-2385 (lower image). The uppermost part shows the voltage-clamp protocol applied. (**B**) Steady-state inactivation curve of *I*_K(DR)_ obtained during exposure to 1 µM PT-2385 (○) or 3 µM PT-2385 (○) (mean ± SEM; *n* = 8 for each point). The continuous lines overlaid on the data points were well-fitted by the Boltzmann equation detailed in Materials and Methods.

When cells were exposed to varying concentrations of PT-2385, the *I*_K(DR)_ activated via membrane depolarization displayed an evident peak, followed by an exponential relaxation to a steady-state level. Hence, we also investigated the quasi-steady-state inactivation curve of *I*_K(DR)_ achieved in the presence of PT-2385 by exploiting a two-step voltage protocol. In this set of current-recording experiments, we placed cells in Ca^2+^-free Tyrode’s solution, and then filled up the pipet with K^+^-enriched solution during the measurements. Once a whole-cell configuration was established, we delivered a two-pulse protocol to the examined cells in situations where varying PT-2385 concentrations were present. According to the least-squares minimization procedure, the best-fitting parameters (i.e., *V*_1/2_ and *q*) for the *I*_K(DR)_-inactivation curve by a test of goodness of fit were convergently obtained in the presence of 1 or 3 µM PT-2385. Consequently, we constructed the normalized amplitude of *I*_K(DR)_ (i.e., *I*/*I*_max_) against the conditioning command potentials (Figure 4A,B), and the continuous sigmoidal curve by adjusting the line to minimize the vertical deviations between the data points, and the line was approximately fitted with a single Boltzmann isotherm, as detailed in Materials and Methods. The values of *V*_1/2_ during exposure to 1 µM and 3 µM PT-2385 were calculated to be −34.7 ± 1.8 and −41.3 ± 1.8 mV (*n* = 8), respectively, while the *q* values (i.e., apparent gating charge) were 2.0 ± 0.3 and 2.1 ± 0.3 *e* (*n* = 8), respectively. These experimental results showed that during exposure to varying PT-2385 concentrations (i.e., 1 and 3 µM), the *V*_1/2_ value of the *I*_K(DR)_-inactivation curve in GH_3_ cells was modified, despite its ineffectiveness in changing the apparent gating charge of the current (i.e., the steepness of the inactivation curve).

### 3.6. PT-2385 on the Recovery of I_K(DR)_ Block Measured from GH_3_ Cells

**Figure 5:**
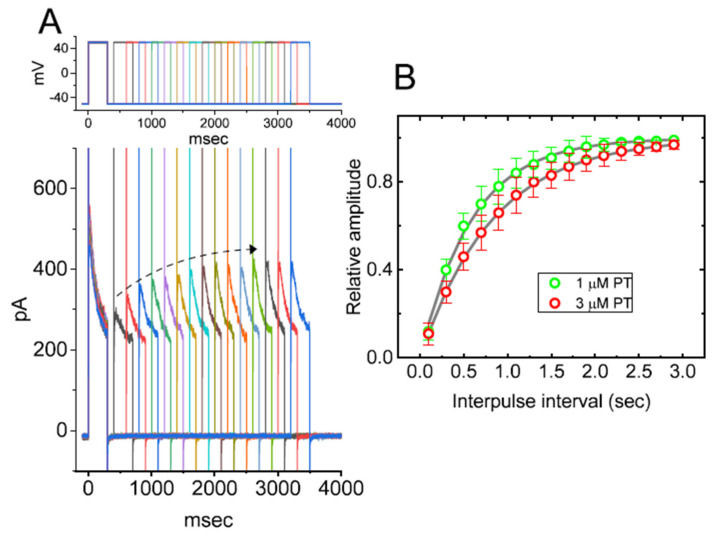
Recovery from *I*_K(DR)_ block caused by PT-2385. In these recording experiments, we kept cells to be bathed in Ca^2+^-free Tyrode’s solution, while the pipet was filled with K^+^-containing solution. The examined GH_3_-cell was depolarized from −50 to +50 mV with a duration of 300 msec, and the different interpulse durations were then delivered. (**A**) Representative *I*_K(DR)_ traces in the presence of 1 µM or 3 µM PT-2385. The dashed curved arrow in (**A**) indicates an exponential rise as a function of the interpulse interval, while the uppermost part denotes the voltage-clamp protocol applied. (**B**) Time course of recovery from *I*_K(DR)_ block taken in the presence of 1 µM PT-2385 (○) or 3 µM PT-2385 (○). The relative amplitude of *I*_K(DR)_ was measured as a ratio of the first peak amplitude divided by the second one. The recovery time course (indicated in gray line) during exposure to 1 or 3 µM PT-2385 was noted to display an exponential rise, with a time constant of 0.59 or 0.83 s, respectively. Each point is the mean ± SEM (*n* = 7 for each point).

We also investigated the recovery from *I*_K(DR)_ block by PT-2385 by the use of another two-step voltage-clamp profile. The voltage-clamp protocol applied consists of an initial (i.e., the first conditioning) depolarizing step from −50 to +50 mV, sufficiently long to ensure the block to reach a steady-state level; subsequently, following different inter-pulse intervals, a second command pulse was applied at the same potential as the first conditioning one (Figure 5A). As the ratios (second current/first current, or relative amplitude) of the peak amplitude of *I*_K(DR)_ activated by the first and the second conditioning pulse were derived for a measure of recovery from *I*_K(DR)_ block in the presence of PT-2385, the value of the recovery time constant was constructed and hence plotted as a function of the inter-pulse interval (Figure 5B). The time course for the recovery of *I*_K(DR)_ block in the presence of 1 or 3 µM PT-2385 was noted to be described by a single-exponential function. By minimizing the squares of the deviations, the time constant for current recovery in the presence of 1 µM PT-2385 was estimated to be 0.59 ± 0.04 s (*n* = 7), significantly smaller than the value of 0.83 ± 0.06 s (*n* = 7, *p* < 0.05) which was obtained during exposure to 3 µM PT-2385. The experimental observations thus tempted us to imply that the slowing of *I*_K(DR)_-current recovery caused by adding PT-2385 would result from its preferential block in the open or open-inactivated state of the K_V_ channel.

### 3.7. Effect of PT-2385 on the Hysteretic Behavior of I_K(DR)_ Triggered by Isosceles-Triangular Ramp Voltage (ITRV) with Varying Durations

**Figure 6:**
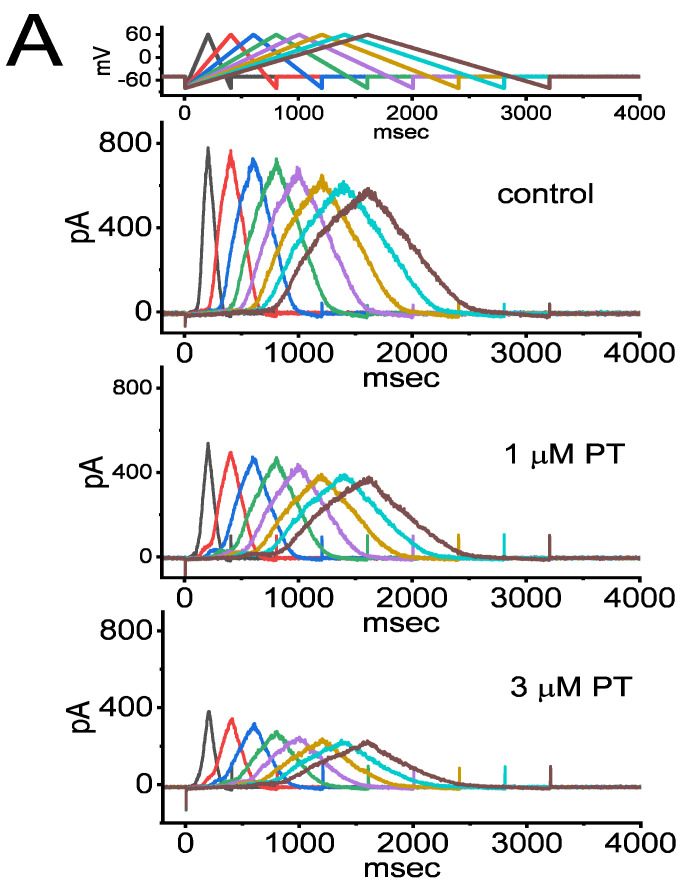

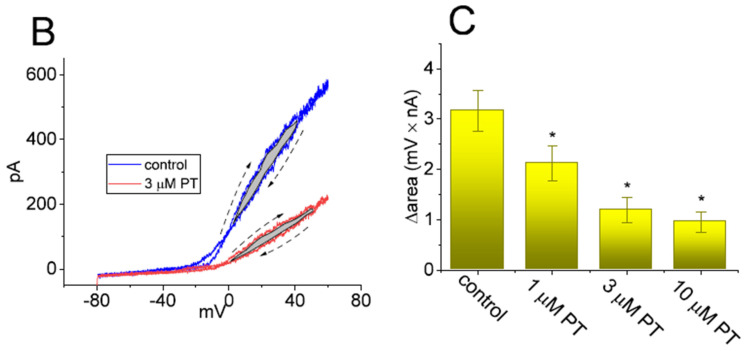
Effect of PT-2385 on *I*_K(DR)_ activated in response to upright isosceles-triangular ramp voltage (ITRV) with varying durations (from 0.4 to 3.2 s), utilized to mimic different depolarizing or repolarizing slopes of bursting patterns in electrically excitable cells. Cells were bathed in Ca^2+^-free Tyrode’s solution containing 1 µM TTX and 0.5 mM CdCl_2_, and the pipet was filled with K^+^-enriched solution. (**A**) Representative current traces activated by the uppermost voltage-clamp ITRV profile, which were obtained in the absence (upper) and presence of 1 µM PT-2385 (middle) or 3 µM PT-2385. Of note, there was a mild decline in peak *I*_K(DR)_ activated by ITRV with increasing ramp-pulse durations. The uppermost part shows the voltage-clamp profile delivered. In (**B**), the effect of PT-2385 (3 µM) on voltage-dependent hysteresis (i.e., the relationship of forward or backward *I*_K(DR)_ versus membrane voltage) activated in response to ITRV with a duration of 3.2 s (or with a ramp speed of ±87.5 mV/s) is illustrated. Current trace in blue color is the control, while that in red was taken after exposure to 3 µM PT-2385. Dashed arrows in (**B**) indicate the direction of *I*_K(DR)_ over which time goes during the elicitation by ITRV. (**C**) Effect of PT-2385 (1, 3, and 10 µM) on the ∆area (i.e., area under the curve activated during the upsloping and downsloping end of ITRV) of voltage-dependent hysteresis elicited by ITRV with a ramp speed of ±87.5 mV/s (mean ± SEM; *n* = 7 for each bar). The shaded area indicates the curve activated during the upsloping and downsloping limb of the upright ITRV. Of note, there was an emergence of voltage-dependent hysteresis for *I*_K(DR)_ activated by ITRV, and the presence of PT-2385 was concentration-dependently able to cause a progressive reduction in the ∆area of voltage hysteresis of the current. * Significantly different from control (i.e., PT-2385 was not present, and currents were evoked by ITRV with ramp speed of ±87.5 mV/s) (*p* < 0.05).

Previous studies have demonstrated the capability of *I*_K(DR)_ strength to influence either varying patterns of bursting firing or action potential configurations in many types of electrically excitable cells [31,37,38,40,41,42,63,64,65]. Therefore, we continued to determine whether and how the presence of PT-2385 could adjust the *I*_K(DR)_ strength activated in response to long-lasting ITRV with varying durations. In this set of experiments, in the absence or presence of PT-2385, we held the examined cell at −50 mV, and an upsloping (forward) limb from −80 to +60 mV followed by a downsloping (backward) limb back to −80 mV (i.e., upright ITRV) with varying durations (between 0.4 and 3.2 s) were thereafter applied (Figure 6A,B). Under this experimental condition, the voltage-dependent hysteresis of *I*_K(DR)_ in response to upright ITRV with varying durations was observed. Of additional interest, during cell exposure to 1 or 3 µM PT-2385, the strength of current responding to both rising and falling limbs of upright ITRV was progressively decreased with increasing the PT-2385 concentrations (Figure 6A). For example, as the duration of isosceles-triangular ramp pulse applied was set at 3.2 s (i.e., the ramp speed of ±87.5 mV/s), the value of ∆area (i.e., the difference in area encircled by the curve in the forward and backward direction) for voltage-dependent hysteresis in the control period (i.e., PT-2385 was not present) was 3.18 ± 0.41 mV·nA (*n* = 8), while at the duration of 3.2 s (i.e., ramp speed at ±87.5 mV/s), the value of ∆area (indicated in shaded area) in the presence of 3 µM PT-2385 was significantly reduced to 1.18 ± 0.25 mV·nA (*n* = 8, *p* < 0.05) (Figure 6C). Findings from these data led us to propose that there was the occurrence of voltage-dependent hysteresis for *I*_K(DR)_ activation in response to upright ITRV in GH_3_ cells [66], and that the hysteretic strength of the current was noticeably reduced with increasing PT-2385 concentration.

### 3.8. Effect of PT-2385 on the Activation Energy Required for I_K(DR)_ Elicitation by ITRV

**Figure 7:**
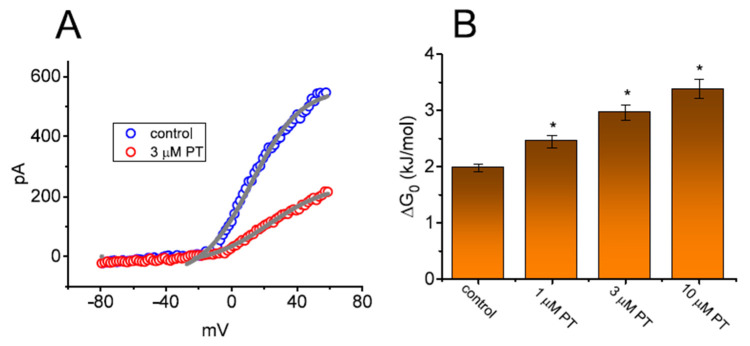
Effect of PT-2385 on the change in free energy at 0 mV (∆G_0_) required for elicitation of *I*_K(DR)_ by ramp-up depolarization. (**A**) Representative instantaneous current traces elicited by the upsloping limb of upright ITRV at a duration of 3.2 s (or with ramp speed of ±87.5 mV/s). The continuous gray lines achieved with or without PT-2385 addition were least-squares fitted to the Boltzmann equation. The values of *V*_1/2_ or *q* (apparent gating charge) activated by upright ITRV with a duration of 3.2 s in the absence (○) and presence (**○**) of 3 µM PT-2385 were estimated to be +12.3 mV or 1.7 *e* and +23.0 mV or 1.3 *e*, respectively. (**B**) The relationship of the free energy level at 0 mV (∆G_0_) versus the different PT-2385 concentrations (mean ± SEM; *n* = 7 for each bar). The estimation of DG_0_ is elaborated under Materials and Methods. Of note, increasing PT-2385 concentrations resulted in higher values of ∆G_0_ identified in GH_3_ cells. * Significantly different from control (*p* < 0.05).

In another set of experiments, we ascertained whether PT-2385 could perturb any modifications in the free energy during *I*_K(DR)_ elicitation caused by ITRV. As illustrated in Figure 7A, in the absence or presence of 3 µM PT-2385, the instantaneous (or transient) activation curve of the current elicited by the upsloping (forward) end of ITRV with a ramp speed of 87.5 mV/s was obtained. Thereafter, the Boltzmann isotherm (detailed in Materials and Methods) was performed with a test of goodness to fit to experimental data points in order to estimate the values of *q* and *V*_1/2_ for such instantaneous activation curves of ITRV-induced *I*_K(DR)_. Based on the values of *q* and *V*_1/2_ during the upsloping end of upright ITRV, the free energy entailed in the gating of *I*_K(DR)_ activation at 0 mV (∆G_0_) in the absence or presence of varying PT-2385 concentrations (i.e., ∆G_0_ = *q* × *F* × *V*_1/2_) was derived and is thereafter illustrated in Figure 7B. Of note, as the PT-2385 concentration was raised, the activation energy (∆G_0_ value) required for *I*_K(DR)_ elicitation was progressively augmented during upright ITRV with a duration of 3.6 s. For example, as GH_3_ cells were exposed to 3 µM PT-2385, the ∆G_0_ value was significantly elevated to 2.98 ± 0.14 kJ/mol from a control value of 1.98 ± 0.07 kJ/mol (*n* = 7, *p* < 0.05).

### 3.9. Effect of PT-2385 on the Hysteresis Activated during Inverted ITRV

**Figure 8:**
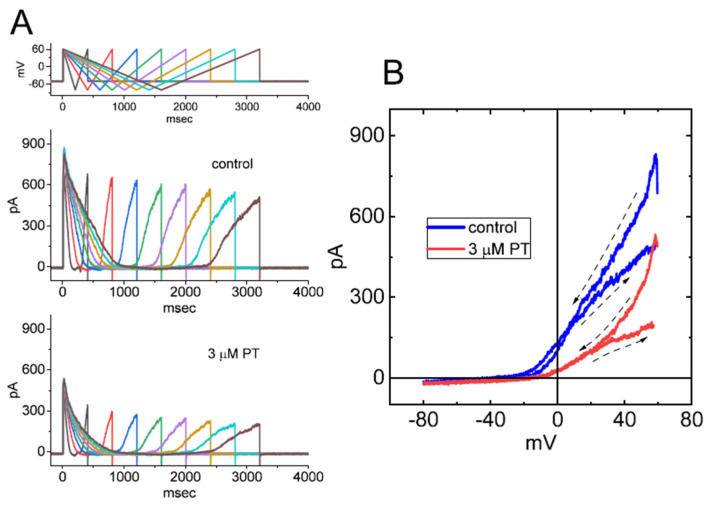
Effect of PT-2385 on hysteresis of *I*_K(DR)_ activated by inverted ITRV in GH_3_ cells. (**A**) Representative current traces obtained in the absence (upper image) and presence of 3 µM PT-2385 (lower image). Current traces were activated by inverted ITRV at varying durations (indicated in the uppermost part). (**B**) Representative hysteresis of *I*_K(DR)_ elicited by inverted ITRV with a ramp speed of ±87.5 mV/s. Dashed arrows in (**B**) indicate the direction of *I*_K(DR)_ over which time goes during the elicitation by ITRV. Of note, the hysteretic behavior of *I*_K(DR)_ in response to inverted ITRV was still observed and was greater than that by upright ITRV, while cell exposure to 3 µM PT-2385 decreased the ∆area of voltage-dependent hysteresis evoked by inverted ITRV.

We continued to determine whether there is hysteresis of *I*_K(DR)_ elicited by inverted ITRV at varying durations. As demonstrated in Figure 8A, in keeping with the above-stated results from upright ITRV, the hysteretic strength of the current by inverted ITRV in the absence and presence of PT-2385 was clearly observed in combination with a gradual decline in peak *I*_K(DR)_ with decreasing ramp speed. As shown in Figure 8B, the relationship of the instantaneous current versus membrane potential was constructed during inverted ITRV with ramp speed of ±87.5 mV/s. Our experimental observations demonstrate the effectiveness of PT-2385 in diminishing the inverted ITRV-induced strength of voltage-dependent hysteresis; meanwhile, the hysteretic magnitude activated by inverted ITRV tends to be stronger than that by upright ITRV with the same ramp speed.

### 3.10. Ability of PT-2385 to Inhibit I_K(DR)_ Amplitude in Human 13-06-MG Glioma Cells

**Figure 9:**
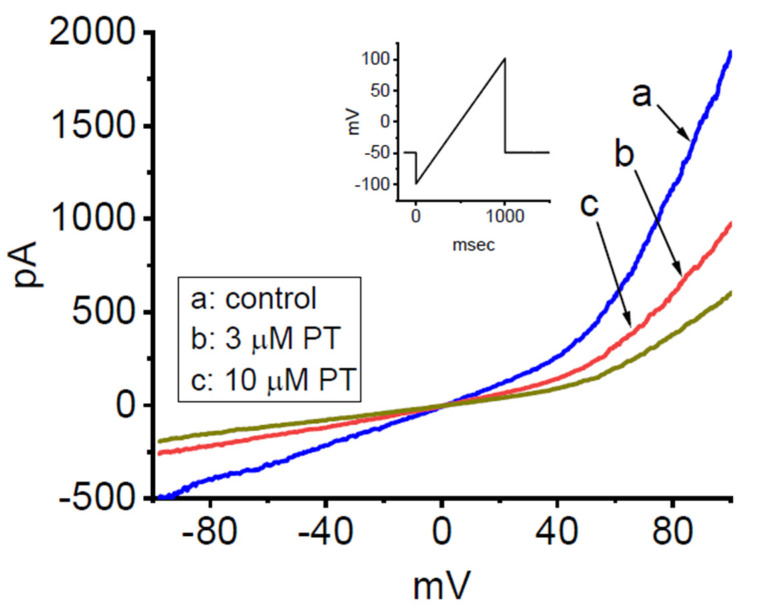
Inhibitory effect of PT-2385 on *I*_K(DR)_ recorded from human 13-06-MG glioma cells. In these experiments, we kept cells to be bathed in high-K^+^, Ca^2+^-free solution and filled up the recording pipet by using a K^+^-enriched solution. The examined cell was voltage-clamped at −50 mV, and an upsloping ramp pulse from −100 to +100 mV for a duration of 1 s was then applied to evoke *I*_K(DR)_. Inset shows the voltage protocol applied. (**a**) control (i.e., PT-2385 was not present); (**b**) 3 µM PT-2385; and (**c**) 10 µM PT-2385.

PT-2385 has been recently reported to inhibit the proliferation of glioma cells [18,19]. In a final stage of experiments, we thus intended to explore whether the presence of PT-2385 was able to modulate ionic currents in other types of neoplastic cells (e.g., malignant glioma cells). As illustrated in Figure 9, in whole-cell current recordings, the magnitude of ramp pulse-induced *I*_K(DR)_ in 13-06-MG glioma cells was robustly detected, as described previously [67]. After 1 min of cell exposure to PT-2385, the amplitude of *I*_K(DR)_ was progressively decreased. For example, at the level of +80 mV, the addition of 3 µM PT-2385 significantly decreased the current amplitude from 1221 ± 228 to 732 ± 187 pA (*n* = 7, *p* < 0.05). After washout of the compound, the amplitude returned to 1217 ± 207 pA (*n* = 7, *p* < 0.05). Therefore, similar to the GH_3_ cells stated above, the *I*_K(DR)_ existing in 13-06-MG cells was noted to be sensitive to inhibition by PT-2385. Apart from the inhibition of HIF-2α, PT-2385-induced changes in malignant behaviors of glioma [18,19] could, to some extent, be closely associated with its inhibitory action of *I*_K(DR)_.

## 4. Discussion

In the present study, cell exposure to PT-2385 was able to decrease the amplitude of *I*_K(DR)_ and raise the inactivation time course of the current. A concentration-dependent inhibition of peak or late *I*_K(DR)_, with respectively effective *IC*_50_ values of 8.1 or 2.2 µM, was observed as GH_3_ cells were exposed to different PT-2385 concentrations. The value of the dissociation constant (*K*_D_) derived from quantitative estimate of the inactivation time course of *I*_K(DR)_ was yielded to be 2.8 µM. Moreover, cell exposure to this compound not only decreased the magnitude of voltage-dependent hysteresis for *I*_K(DR)_ in reply to upright or inverted isosceles-triangular ramp voltage (ITRV), but also increased free energy involved in the gating *I*_K(DR)_ elicitation. It could also decrease the amplitude of *I*_K(DR)_ in human 13-06-MG glioma cells. Collectively, the PT-2385-mediated effectiveness in the modifications of ionic currents described herein tends to be independent of an interaction with HIF-2α and it could thus be a confounding factor affecting neuronal or endocrine function.

Perhaps more noticeable with respect to the issue of the magnitude in the PT-2385-mediated block of *I*_K(DR)_ observed in GH_3_ cells is that the rising phase of the current activated in response to rapid membrane depolarization remained little-altered in its presence; however, the addition of this compound had the propensity to accelerate *I*_K(DR)_ inactivation in a time-, state-, and concentration-dependent fashion. This could thus be interpreted to mean that PT-2385 is able to reach the blocking site in situations where the K_V_ channel favorably resides in the open or open-inactivated state. Consequently, this property is reasonably explained by the first-order binding scheme (i.e., closed↔open↔open·blocked), as demonstrated above [30,59]. Pertinent to such reaction scheme is that open-blocked K_V_ channels are not closed unless the PT-2385 dissociates from the binding site. Meanwhile, the time-dependent block produced by PT-2385 reflects that it preferentially binds to and blocks an open state (or conformation) of the channel with a *K*_D_ value of 2.9 µM observed in GH_3_ cells, a value which is close to the *IC*_50_ value (i.e., 2.2 µM) entailed for its inhibitory effect on late *I*_K(DR)_, not on peak *I*_K(DR)_.

The magnitude of *I*_K(DR)_ reduced by PT-2385, concomitantly with a considerable rise in current inactivation in response to long sustained depolarization, may be the cause of its perturbing effects on membrane excitability in varying types of electrically excitable cells (e.g., neurons and neuroendocrine or endocrine cells). Because the recovery from current inactivation in the presence of PT-2385 observed in this study also became slowed, a duration greater than 3 s was required for current magnitude to recover completely. The steady-state inactivation curve of *I*_K(DR)_ was more hyperpolarized as the PT-2385 concentration was increased. As a consequence, a PT-2385-perturbed block of *I*_K(DR)_ will even become pronounced when a train of action potentials occurs, since the *I*_K(DR)_ magnitude, especially at the occurrence of transient resurgent K^+^ currents, is decreased as a function of firing frequency [32,40,41,42].

Recent pharmacokinetic studies have shown that after being administered orally at twice-per-day dose of 800 mg, the plasma level of PT-2385 in patients with clear cell renal cell carcinoma was reported to reach around 3000–5000 ng/mL (or 7.8–13.0 µM) [20]. Therefore, it is possible that the used concentration of PT-2385 in this study tends to be achievable in the plasma of treated patients. Any changes in the amplitude, gating, and hysteresis of *I*_K(DR)_ caused by PT-2385 depend not simply on the PT-2385 concentration, but also on the preexisting resting potential and varying bursting patterns of excitable cells. From this perspective, the observed effects by PT-2385 in this study are likely to arise to the extent of clinically achievable concentrations.

There seems to be a divergence in the *IC*_50_ value between the PT-2385-induced block of late *I*_K(DR)_ (around 2.2 µM) and its effect on HIF-2α inhibition (around 27 nM) [22]. The plausible interpretation for this difference is unknown at present; however, it could be due to the varying experimental maneuvers used in the study. In terms of investigations on HIF-2α inhibition, surface-membrane components in host cells could have mostly been lysed and removed; consequently, PT-2385 could readily reach HIF-2α inside the cells [13,14,22]. However, in the present observations, intact cells were used to investigate different types of membrane ionic currents in GH_3_ cells or 13-06-MG glioma cells.

Under hypoxic conditions along with possible changes in HIF expression, the activity of ATP-dependent K^+^ (K_ATP_) channels in GH_3_ cells or 13-06-MG glioma cells could be elevated during exposure to PT-2385 [47,48,49,50]. The presence of PT-2385 was previously demonstrated to counteract the adverse effect of sorafenib (SOR), known to suppress the activity of tyrosine kinases, in hepatocellular carcinoma [16]. However, in our whole-cell current recording, the intracellular solution contained 3 mM ATP, a value known to adequately suppress K_ATP_-channel activity [60,68]. However, the PT-2385-mediated inhibition of *I*_K(DR)_ observed in GH_3_ cells was little -affected by the subsequent application of either 30 µM diazoxide (Diaz) or 10 µM cilostazol (Cil). Diaz or Cil were respectively reported to be stimulators of K_ATP_ channels or large-conductance Ca^2+^-activated K^+^ (BK_Ca_) channels [34,60,61]. Moreover, in the continued presence of PT-2385, the subsequent addition of sorafenib (SOR), an inhibitor of tyrosine kinase [62], failed to modify PT-2385-induced block of *I*_K(DR)_ in GH_3_ cells. As such, a PT-induced reduction in whole-cell *I*_K(DR)_ strength depicted in recent study is unlikely to be linked either to inhibition of K_ATP_- or BK_Ca_-channel activity, or to stimulation of tyrosine-kinase activity, although these channels or enzymes are functionally active in pituitary cells [60,61,62].

It needs to be emphasized that as it is administered, PT-2385 needs to enter cytosol or even the nucleus before being able interact with HIF-2α or the HIF-α/HIF-β complex [12,13,14,21]. In other words, despite being small in size, the molecules must pass through cell-surface membrane before entering the cell interior and then reaching the target. In this regard, it is reasonable to assume that the perturbations to plasmalemmal ionic currents caused by PT-2385 would precede its subsequent actions through the inhibition of HIF-2α. The extent of PT-2385-mediated modifications to ionic currents (e.g., *I*_K(DR)_) is closely linked to the inhibition of HIF-2α, followed by changes in proliferation of different types of neoplastic cells [12,13,14,16,17,26].

The perturbations by PT-2385 of membrane ionic currents shown here tend to be acute in onset. Hence, the actions could be unlinked to its interaction with the activity of HIF-2α, as stated recently [9,12,14,25]. Both the steady-state inactivation curve of *I*_K(DR)_ and recovery from current inactivation were demonstrated in the presence of PT-2385. The actions of this compound on the magnitude or kinetic gating of *I*_K(DR)_ are thought to occur either through its preferential binding to the open state of the K_V_ channel or through an interaction with the channel’s open conformation.

The phenomenon of the voltage-dependent hysteresis of ionic currents has been proposed to play roles in affecting the electrical behavior of electrically excitable cells. Consistent with previous observations on other types of ionic currents [45,52,57,66], the *I*_K(DR)_ present in GH_3_ cells underwent hysteresis in which there is likely to be a mode shift, because the voltage sensitivity of the gating charge movement is thought to depend on the previous state of the channel [57,66]. Moreover, we further evaluated the possible perturbation by PT-2385 of the instantaneous and non-equilibrium property inherent in *I*_K(DR)_ activated by upright or inverted ITRV. The results found PT-2385′s effectiveness in decreasing the ∆area of hysteresis for *I*_K(DR)_ elicited by ITRV. Moreover, the presence of PT-2385 could increase the ∆G_0_ value for *I*_K(DR)_ activation in response to an instantaneous upsloping ramp pulse. In this regard, any modifications of *I*_K(DR)_ caused by PT-2385 depend not only on the PT-2385 concentration used but also various factors that include the pre-existing resting potential or varying firing patterns of action potentials.

It is important to mention that a possible link between HIF and regulation of K_V_-channel activity has been demonstrated [23,47,48,49,50]. Another study [69] showed the ability of HIF-1 to regulate K_V_1.2 channels in rat PC12 cells. It is possible that HIF-2α regulates K_V_-channel activity directly. Whether PT-2385 exercises an inhibitory effect on *I*_K(DR)_ in GH_3_ or 13-05-MG cells where HIF-2α is knocked down remains to be further investigated.

All in all, in this study, we hitherto revealed the unidentified action of PT-2385 through which it could modify the amplitude, gating, and hysteretic behavior of *I*_K(DR)_. The modifications to the magnitude, gating, and voltage-dependent hysteresis of *I*_K(DR)_ caused by the presence of PT-2385 are not simply a bystander effect, and may potentially converge to act on the functional activities of neurons and endocrine or neuroendocrine cells if similar in vivo observations occur. To what extent such direct actions on *I*_K(DR)_ contribute to the rapid impairment in hypoxic ventilatory drive [62] remains to be further resolved.

## Data Availability

Not applicable.

## Supplementary Materials

The following are available online at https://www.mdpi.com/article/10.3390/membranes11080636/s1, The preparation of GH3 or 13-06-MG is reported in the Supplementary Materials. References [51,52] are cited in the Supplementary Materials.

## Abbreviations

Cil	cilostazol
Diaz	Diazoxide
∆G_0_	free energy involved in current activation at 0 mV
HIF	hypoxia-inducible factor
*I-V*	current versus voltage
*IC*_50_	the concentration required for 50% inhibition
*I*_K(DR)_	delayed-rectifier K^+^ current
ITRV	isosceles-triangular ramp voltage
K_ATP_	channel, ATP-sensitive K^+^ channel
*K*_D_	dissociation constant
K_V_	channel, voltage-gated K^+^ channel
SEM	standard error of mean
SOR	Sorafenib
TEA	tetraethylammonium chloride
τ_inact(S)_	time constant in the slow component of current inactivation
TTX	tetrodotoxin

## References

[1] Vidal S., Horvath E., Kovacs K., Kuroki T., Lloyd R.V., Scheithauer B.W. (2003). Expression of hypoxia-inducible factor-1alpha (HIF-1alpha) in pituitary tumours. Histol. Histopathol..

[2] Kim K., Yoshida D., Teramoto A. (2005). Expression of Hypoxia-Inducible Factor 1α and Vascular Endothelial Growth Factor in Pituitary Adenomas. Endocr. Pathol..

[3] Yoshida D., Kim K., Noha M., Teramoto A. (2006). Anti-Apoptotic Action by Hypoxia Inducible Factor 1-Alpha in Human Pituitary Adenoma Cell Line, HP-75 in Hypoxic Condition. J. Neuro Oncol..

[4] Yoshida D., Kim K., Yamazaki M., Teramoto A. (2005). Expression of Hypoxia-Inducible Factor 1α and Cathepsin D in Pituitary Adenomas. Endocr. Pathol..

[5] Yoshida D., Koketshu K., Nomura R., Teramoto A. (2010). The CXCR4 antagonist AMD3100 suppresses hypoxia-mediated growth hormone production in GH3 rat pituitary adenoma cells. J. Neuro Oncol..

[6] Nomura R., Yoshida D., Teramoto A. (2009). Stromal cell-derived factor-1 expression in pituitary adenoma tissues and upregulation in hypoxia. J. Neuro Oncol..

[7] Lei T., Xiao Z., Liu Q., Zhao B., Wu J. (2011). Hypoxia induces hemorrhagic transformation in pituitary adenomas via the HIF-1? signaling pathway. Oncol. Rep..

[8] Zhang C., Qiang Q., Jiang Y., Hu L., Ding X., Lu Y., Hu G. (2015). Effects of hypoxia inducible factor-1α on apoptotic inhibition and glucocorticoid receptor downregulation by dexamethasone in AtT-20 cells. BMC Endocr. Disord..

[9] Kinali B., Senoglu M., Karadag F.K., Karadag A., Middlebrooks E.H., Oksuz P., Sandal E., Turk C., Diniz G. (2019). Hypoxia-Inducible Factor 1α and AT-Rich Interactive Domain-Containing Protein 1A Expression in Pituitary Adenomas: Association with Pathological, Clinical, and Radiological Features. World Neurosurg..

[10] Lucia K., Wu Y., Garcia J.M., Barlier A., Buchfelder M., Saeger W., Renner U., Stalla G.K., Theodoropoulou M. (2020). Hypoxia and the hypoxia inducible factor 1α activate protein kinase A by repressing RII beta subunit transcription. Oncogene.

[11] Tella S.H., Taïeb D., Pacak K. (2017). HIF-2alpha: Achilles’ heel of pseudohypoxic subtype paraganglioma and other related conditions. Eur. J. Cancer.

[12] Chen W., Hill H., Christie A., Kim M.S., Holloman E., Pavia-Jimenez A., Homayoun F., Ma Y., Patel N., Yell P. (2016). Targeting renal cell carcinoma with a HIF-2 antagonist. Nat. Cell Biol..

[13] Wallace E.M., Rizzi J.P., Han G., Wehn P.M., Cao Z., Du X., Cheng T., Czerwinski R.M., Dixon D.D., Goggin B.S. (2016). A Small-Molecule Antagonist of HIF2α Is Efficacious in Preclinical Models of Renal Cell Carcinoma. Cancer Res..

[14] Martínez-Sáez O., Borau P.G., Alonso-Gordoa T., Molina-Cerrillo J., Grande E. (2017). Targeting HIF-2 α in clear cell renal cell carcinoma: A promising therapeutic strategy. Crit. Rev. Oncol..

[15] Xie C., Yagai T., Luo Y., Liang X., Chen T., Wang Q., Sun D., Zhao J., Ramakrishnan S.K., Sun L. (2017). Activation of intestinal hypoxia-inducible factor 2α during obesity contributes to hepatic steatosis. Nat. Med..

[16] Xu J., Zheng L., Chen J., Sun Y., Lin H., Jin R.-A., Tang M., Liang X., Cai X. (2017). Increasing AR by HIF-2α inhibitor (PT-2385) overcomes the side-effects of sorafenib by suppressing hepatocellular carcinoma invasion via alteration of pSTAT3, pAKT and pERK signals. Cell Death Dis..

[17] Yoshino H., Nohata N., Miyamoto K., Yonemori M., Sakaguchi T., Sugita S., Itesako T., Kofuji S., Nakagawa M., Dahiya R. (2017). PHGDH as a Key Enzyme for Serine Biosynthesis in HIF2α-Targeting Therapy for Renal Cell Carcinoma. Cancer Res..

[18] Renfrow J.J., Soike M.H., Debinski W., Ramkissoon S.H., Mott R.T., Frenkel M.B., Sarkaria J.N., Lesser G.J., E Strowd R. (2018). Hypoxia-inducible factor 2α: A novel target in gliomas. Futur. Med. Chem..

[19] Renfrow J.J., Soike M.H., West J.L., Ramkissoon S.H., Metheny-Barlow L., Mott R.T., Kittel C.A., D’Agostino R.B., Tatter S.B., Laxton A.W. (2020). Attenuating hypoxia driven malignant behavior in glioblastoma with a novel hypoxia-inducible factor 2 alpha inhibitor. Sci. Rep..

[20] Courtney K.D., Infante J.R., Lam E.T., Figlin R.A., Rini B.I., Brugarolas J., Zojwalla N.J., Lowe A.M., Wang K., Wallace E.M. (2018). Phase I Dose-Escalation Trial of PT2385, a First-in-Class Hypoxia-Inducible Factor-2α Antagonist in Patients With Previously Treated Advanced Clear Cell Renal Cell Carcinoma. J. Clin. Oncol..

[21] Courtney K.D., Ma Y., de Leon A.D., Christie A., Xie Z., Woolford L., Singla N., Joyce A., Hill H., Madhuranthakam A.J. (2020). HIF-2 Complex Dissociation, Target Inhibition, and Acquired Resistance with PT2385, a First-in-Class HIF-2 Inhibitor, in Patients with Clear Cell Renal Cell Carcinoma. Clin. Cancer Res..

[22] Wehn P.M., Rizzi J.P., Dixon D.D., Grina J.A., Schlachter S.T., Wang B., Xu R., Yang H., Du X., Han G. (2018). Design and Activity of Specific Hypoxia-Inducible Factor-2α (HIF-2α) Inhibitors for the Treatment of Clear Cell Renal Cell Carcinoma: Discovery of Clinical Candidate (S)-3-((2,2-Difluoro-1-hydroxy-7-(methylsulfonyl)-2,3-dihydro-1*H*-inden-4-yl)oxy)-5-fluorobenzonitrile (PT2385). J. Med. Chem..

[23] Qian C., Dai Y., Xu X., Jiang Y. (2019). HIF-1α Regulates Proliferation and Invasion of Oral Cancer Cells through Kv3.4 Channel. Ann. Clin. Lab Sci..

[24] Schwartz A.J., Das N.K., Ramakrishnan S.K., Jain C., Jurkovic M.T., Wu J., Nemeth E., Lakhal-Littleton S., Colacino J.A., Shah Y.M. (2018). Hepatic hepcidin/intestinal HIF-2α axis maintains iron absorption during iron deficiency and overload. J. Clin. Investig..

[25] Hsu T.-S., Lin Y.-L., Wang Y.-A., Mo S.-T., Chi P.-Y., Lai A.C.-Y., Pan H.-Y., Chang Y.-J., Lai M.-Z. (2020). HIF-2α is indispensable for regulatory T cell function. Nat. Commun..

[26] Persson C.U., von Stedingk K., Fredlund E., Bexell D., Påhlman S., Wigerup C., Mohlin S. (2020). ARNT-dependent HIF-2 transcriptional activity is not sufficient to regulate downstream target genes in neuroblastoma. Exp. Cell Res..

[27] Chang W.-T., Lo Y.-C., Gao Z.-H., Wu S.-N. (2019). Evidence for the Capability of Roxadustat (FG-4592), an Oral HIF Prolyl-Hydroxylase Inhibitor, to Perturb Membrane Ionic Currents: An Unidentified yet Important Action. Int. J. Mol. Sci..

[28] Wulfsen I., Hauber H.P., Schiemann D., Bauer C.K., Schwarz J.R. (2001). Expression of mRNA for voltage-dependent and inward-rectifying K channels in GH3/B6 cells and rat pituitary. J. Neuroendocr..

[29] Tsai T.-Y., Tsai Y.-C., Wu S.-N., Liu Y.-C. (2006). Tramadol-induced blockade of delayed rectifier potassium current in NG108-15 neuronal cells. Eur. J. Pain.

[30] Wang Y.-J., Lin M.-W., Lin A.-A., Peng H., Wu S.-N. (2008). Evidence for state-dependent block of DPI 201-106, a synthetic inhibitor of Na+ channel inactivation, on delayed-rectifier K+ current in pituitary tumor (GH3) cells. J. Physiol. Pharmacol. Off. J. Pol. Physiol. Soc..

[31] Huang C.-C., Tsai J.J., Wu S.N. (2009). Experimental and simulation studies on the mechanisms of levetiracetam-mediated inhibition of delayed-rectifier potassium current (KV3.1): Contribution to the firing of action potentials. J. Physiol. Pharmacol. Off. J. Pol. Physiol. Soc..

[32] Kaczmarek L.K., Zhang Y. (2017). Kv3 Channels: Enablers of Rapid Firing, Neurotransmitter Release, and Neuronal Endurance. Physiol. Rev..

[33] Fletcher P.A., Sherman A., Stojilkovic S.S. (2018). Common and diverse elements of ion channels and receptors underlying electrical activity in endocrine pituitary cells. Mol. Cell. Endocrinol..

[34] Kuo P.-C., Yang C.-J., Lee Y.-C., Chen P.-C., Liu Y.-C., Wu S.-N. (2018). The comprehensive electrophysiological study of curcuminoids on delayed-rectifier K + currents in insulin-secreting cells. Eur. J. Pharmacol..

[35] Hernández-Pineda R., Chow A., Amarillo Y., Moreno H., Saganich M., De Miera E.C.V.-S., Hernández-Cruz A., Rudy B. (1999). Kv3.1–Kv3.2 Channels Underlie a High-Voltage–Activating Component of the Delayed Rectifier K+ Current in Projecting Neurons From the Globus Pallidus. J. Neurophysiol..

[36] Schultz J.-H., Volk T., Ehmke H. (2001). Heterogeneity of Kv2.1 mRNA expression and delayed rectifier current in single isolated myocytes from rat left ventricle. Circ. Res..

[37] Baranauskas G. (2007). Ionic Channel Function in Action Potential Generation: Current Perspective. Mol. Neurobiol..

[38] Johnston J., Griffin S.J., Baker C., Skrzypiec A., Chernova T., Forsythe I. (2008). Initial segment Kv2.2 channels mediate a slow delayed rectifier and maintain high frequency action potential firing in medial nucleus of the trapezoid body neurons. J. Physiol..

[39] Bocksteins E., Van De Vijver G., Van Bogaert P.-P., Snyders D.J. (2012). Kv3 channels contribute to the delayed rectifier current in small cultured mouse dorsal root ganglion neurons. Am. J. Physiol. Physiol..

[40] Liu P.W., Bean B.P. (2014). Kv2 Channel Regulation of Action Potential Repolarization and Firing Patterns in Superior Cervical Ganglion Neurons and Hippocampal CA1 Pyramidal Neurons. J. Neurosci..

[41] Labro A.J., Priest M.F., Lacroix J.J., Snyders D.J., Bezanilla F. (2015). Kv3.1 uses a timely resurgent K+ current to secure action potential repolarization. Nat. Commun..

[42] Brown M.R., El-Hassar L., Zhang Y., Alvaro G., Large C.H., Kaczmarek L.K. (2016). Physiological modulators of Kv3.1 channels adjust firing patterns of auditory brain stem neurons. J. Neurophysiol..

[43] Chao R.-Y., Cheng C.-H., Wu S.-N., Chen P.-C. (2017). Defective trafficking of Kv2.1 channels in MPTP-induced nigrostriatal degeneration. J. Neurochem..

[44] Di Lucente J., Nguyen H.M., Wulff H., Jin L.-W., Maezawa I. (2018). The voltage-gated potassium channel Kv1.3 is required for microglial pro-inflammatory activation in vivo. Glia.

[45] Lu T.-L., Lu T.-J., Wu S.-N. (2020). Inhibitory effective perturbations of cilobradine (dk-ah269), a blocker of hcn channels, on the amplitude and gating of both hyperpolarization-activated cation and delayed-rectifier potassium currents. Int. J. Mol. Sci..

[46] Cheng X., Prange-Barczynska M., Fielding J.W., Zhang M., Burrell A.L., Lima J.D., Eckardt L., Argles I.L., Pugh C.W., Buckler K.J. (2020). Marked and rapid effects of pharmacological HIF-2α antagonism on hypoxic ventilatory control. J. Clin. Investig..

[47] Raeis V., Philip-Couderc P., Roatti A., Habre W., Sierra J., Kalangos A., Beghetti M., Baertschi A.J. (2010). Central venous hypoxemia is a determinant of human atrial atp-sensitive potassium channel expression: Evidence for a novel hypoxia-inducible factor 1alpha-forkhead box class o signaling pathway. Hypertension.

[48] Zhang C.C., Sadek H.A. (2014). Hypoxia and Metabolic Properties of Hematopoietic Stem Cells. Antioxid. Redox Signal..

[49] Yu L., Li W., Park B.M., Lee G.-J., Kim S.H. (2019). Hypoxia augments nahs-induced anp secretion via katp channel, hif-1α and ppar-γ pathway. Peptides.

[50] Li J., Zhou W., Chen W., Wang H., Zhang Y., Yu T. (2020). Mechanism of the hypoxia inducible factor 1/hypoxic response element pathway in rat myocardial ischemia/diazoxide post-conditioning. Mol. Med. Rep..

[51] Huang M.-H., Liu P.-Y., Wu S.-N. (2019). Characterization of Perturbing Actions by Verteporfin, a Benzoporphyrin Photosensitizer, on Membrane Ionic Currents. Front. Chem..

[52] Lo Y.-C., Lin C.-L., Fang W.-Y., Lőrinczi B., Szatmári I., Chang W.-H., Fülöp F., Wu S.-N. (2021). Effective Activation by Kynurenic Acid and Its Aminoalkylated Derivatives on M-Type K^+^ Current. Int. J. Mol. Sci..

[53] Wu S.-N., Hsu M.-C., Liao Y.-K., Wu F.-T., Jong Y.-J., Lo Y.-C. (2012). Evidence for inhibitory effects of flupirtine, a centrally acting analgesic, on delayed rectifier k(+) currents in motor neuron-like cells. Evid. Based Complement Altern. Med..

[54] Yifrach O., MacKinnon R. (2002). Energetics of Pore Opening in a Voltage-Gated K+ Channel. Cell.

[55] Wu S.-N., Yeh C.-C., Huang H.-C., Yang W.-H. (2011). Cholesterol depletion with (2-hydroxypropyl)- beta-cyclodextrin modifies the gating of membrane electroporation-induced inward current in pituitary tumor gh3 cells: Experimental and analytical studies. Cell Physiol. Biochem..

[56] Hsu H.-T., Lo Y.-C., Huang Y.-M., Tseng Y.-T., Wu S.-N. (2017). Important modifications by sugammadex, a modified γ-cyclodextrin, of ion currents in differentiated NSC-34 neuronal cells. BMC Neurosci..

[57] Villalba-Galea C.A., Chiem A.T. (2020). Hysteretic Behavior in Voltage-Gated Channels. Front. Pharmacol..

[58] Vivaudou M. (2019). eeFit: A Microsoft Excel-embedded program for interactive analysis and fitting of experimental dose–response data. Biotech..

[59] Lin M.-W., Wang Y.-J., Liu S.-I., Lin A.-A., Lo Y.-C., Wu S.-N. (2008). Characterization of aconitine-induced block of delayed rectifier K+ current in differentiated NG108-15 neuronal cells. Neuropharmacology.

[60] Wu S.-N., Li H.-F., Chiang H.-T. (2000). Characterization of ATP-sensitive potassium channels functionally expressed in pituitary GH3 cells. J. Membr. Biol..

[61] Wu S.-N., Liu S.-I., Huang M.-H. (2004). Cilostazol, an inhibitor of type 3 phosphodiesterase, stimulates large-conductance, calcium-activated potassium channels in pituitary gh3 cells and pheochromocytoma pc12 cells. Endocrinology.

[62] Chang W.-T., Liu P.-Y., Lee K., Feng Y.-H., Wu S.-N. (2020). Differential Inhibitory Actions of Multitargeted Tyrosine Kinase Inhibitors on Different Ionic Current Types in Cardiomyocytes. Int. J. Mol. Sci..

[63] Delmar M., Ibarra J., Davidenko J., Lorente P., Jalife J. (1991). Dynamics of the background outward current of single guinea pig ventricular myocytes. Ionic mechanisms of hysteresis in cardiac cells. Circ. Res..

[64] Kusters J.M.A.M., Cortes J.M., Van Meerwijk W.P.M., Ypey D.L., Theuvenet A.P.R., Gielen C.C.A.M. (2007). Hysteresis and Bistability in a Realistic Cell Model for Calcium Oscillations and Action Potential Firing. Phys. Rev. Lett..

[65] Wu S.-N., Chen B.-S., Lin M.-W., Liu Y.-C. (2008). Contribution of slowly inactivating potassium current to delayed firing of action potentials in NG108-15 neuronal cells: Experimental and theoretical studies. J. Theor. Biol..

[66] Villalba-Galea C.A. (2016). Hysteresis in voltage-gated channels. Channels.

[67] Huang M.H., Huang Y.-M., Wu S.-N. (2015). The inhibition by oxaliplatin, a platinum-based anti-neoplastic agent, of the activity of intermediate-conductance ca²⁺-activated k⁺ channels in human glioma cells. Cell Physiol. Biochem..

[68] Wu S.-N., Wu A.Z., Sung R.J. (2007). Identification of two types of ATP-sensitive K+ channels in rat ventricular myocytes. Life Sci..

[69] Dong Q., Zhao N., Xia C.-K., Du L.-L., Fu X.-X., Du Y.-M. (2012). Hypoxia induces voltage-gated K+ (Kv) channel expression in pulmonary arterial smooth muscle cells through hypoxia-inducible factor-1 (HIF-1). Bosn. J. Basic Med. Sci..

