# Supplementary material for: Effective Perturbations of the Amplitude, Gating, and Hysteresis of IK(DR) Caused by PT-2385, an HIF-2α Inhibitor
Source: Membranes (Basel). 2021 Aug 17;11(8):636. doi: 10.3390/membranes11080636 (PMC8398179; doi:10.3390/membranes11080636)
Supplement: Supplementary file 1 [file membranes-11-00636-s001.zip › membranes-1301050-supplementary.pdf]

membranes

Supplementary Information

Effective Perturbations of the Amplitude, Gating, and Hysteresis of $I_{K(DR)}$ Caused by PT-2385, an HIF-2α Inhibitor

Hung-Tsung Hsiao [1], Guan-Ling Lu [1], Yen-Chin Liu [1,2,*] and Sheng-Nan Wu [3,4,*]

1 Department of Anesthesiology, National Cheng Kung University Hospital, College of Medicine, National Cheng Kung University, Tainan City 70403, Taiwan; aneshsiao@yahoo.com.tw (H.-T.H.); r93443013@ntu.edu.tw (G.-L.L.)
2 Department of Anesthesiology, School of Post-Baccalaureate, College of Medicine, Kaohsiung Medical University, Kaohsiung City 80708, Taiwan
3 Institute of Basic Medical Sciences, College of Medicine, National Cheng Kung University, Tainan City 70101, Taiwan
4 Department of Physiology, College of Medicine, National Cheng Kung University, Tainan City 70101, Taiwan
* Correspondence: anesliu@gmail.com (Y.-C.L.); snwu@mail.ncku.edu.tw (S.-N.W.); Tel.: +886-(623)-5353-55334 (S.-N.W.); Fax: 886-6-2362780 (S.-N.W.)

Citation: Hsiao, H.-T.; Lu, G.-L.; Liu, Y.-C.; Wu, S.-N. Effective Perturbations of the Amplitude, Gating and Hysteresis of $I_{K(DR)}$ Caused by PT-2385, an HIF-2α Inhibitor. *Membranes* **2021**, *11*, 636. https://doi.org/10.3390/membranes11080636

Academic Editor: Julia Schumann

Received: 30 June 2021
Accepted: 10 August 2021
Published: 17 August 2021

Publisher's Note: MDPI stays neutral with regard to jurisdictional claims in published maps and institutional affiliations.

Copyright: © 2021 by the authors. Licensee MDPI, Basel, Switzerland. This article is an open access article distributed under the terms and conditions of the Creative Commons Attribution (CC BY) license (http://creativecommons.org/licenses/by/4.0/).

Cell preparations used in the present work

GH_3 was acquired from the Bioresources Collection and Research Center ([BCRC-60015]; Hsinchu, Taiwan) and originally derived from prolactin-secreting pituitary tumor of the rat. Briefly, cells were cultured with Ham's F-12 medium contained supplement of 15% (v/v) horse serum, 2 mM L-glutamine and 2.5% (v/v) fetal calf serum [1]. GH_3 cells were transferred to a serum-free Ca^{2+}-free medium to promote cell differentiation. The multiforme cell line of glioblastoma (13-06-MG) was grown in high-glucose (4 g/L) Dulbecco's modified Eagle media supplemented with 10% (v/v) fetal bovine serum and was provided kindly by Professor Dr. Carol A. Kruse (Department of Neurosurgery, Ronald Reagan UCLA Medical Center, LA, CA)[2]. GH_3 or 13-06-MG cells were maintained in a humidified environment of 5% CO_2/95% air at 37 °C. By identifying glial fibrillary acidic protein, a cytoskeletal protein, glial cells were verified.

References

1. Huang, M.H.; Liu, P.Y.; Wu, S.N. Characterization of perturbing actions by verteporfin, a benzoporphyrin photosensitizer, on membrane ionic currents. *Front Chem* **2019**, *7*, 566.
2. Lo, Y.C.; Lin, C.L.; Fang, W.Y.; Lőrinczi, B.; Szatmári, I. Effective activation by kynurenic acid and its aminoalkylated derivatives on m-type k(+) current. **2021**, *22*.
